# Advances in the clinical application of machine learning in acute pancreatitis: a review

**DOI:** 10.3389/fmed.2024.1487271

**Published:** 2025-01-07

**Authors:** Zhaowang Tan, Gaoxiang Li, Yueliang Zheng, Qian Li, Wenwei Cai, Jianfeng Tu, Senjun Jin

**Affiliations:** Emergency and Critical Care Center, Department of Emergency Medicine, Zhejiang Provincial People’s Hospital, People’s Hospital of Hangzhou Medical College, Hangzhou, Zhejiang, China

**Keywords:** artificial intelligence, machine-learning model, acute pancreatitis, severity, complications, recurrence, mortality

## Abstract

Traditional disease prediction models and scoring systems for acute pancreatitis (AP) are often inadequate in providing concise, reliable, and effective predictions regarding disease progression and prognosis. As a novel interdisciplinary field within artificial intelligence (AI), machine learning (ML) is increasingly being applied to various aspects of AP, including severity assessment, complications, recurrence rates, organ dysfunction, and the timing of surgical intervention. This review focuses on recent advancements in the application of ML models in the context of AP.

## 1 Introduction

Acute pancreatitis (AP) is an inflammatory disorder affecting the parenchyma and peripancreatic tissue, characterized by severe abdominal pain, elevated pancreatic enzymes, and pancreatitis-related changes on abdominal imaging. The incidence of AP has shown a rising trend globally, with an average occurrence rate of 34 cases per 100,000 individuals. Approximately 20% of patients progress to either moderately severe acute pancreatitis (MSAP, accompanied by transient [≤48 h] organ dysfunction and/or local complications such as necrosis of pancreatic or peripancreatic tissue) or severe acute pancreatitis (SAP, accompanied by persistent [>48 h] organ failure), the mortality rate can reach as high as 20–40% (1).

Machine learning (ML) is a category of artificial intelligence tools in which virtual agents learn an optimized set of rules through trial and error—a policy that maximizes expected returns (2). ML has many ideal characteristics that can help with medical decision-making, and these algorithms are able to infer the best decision from suboptimal training sets. ML has been successfully applied to medical problems in the past, such as diabetes and sepsis (3, 4).

Machine learning has demonstrated significant potential in the field of medicine, particularly in disease diagnosis and prognosis. Over the past decade, the utilization of ML algorithms based on databases for acute pancreatitis has become increasingly prevalent. Numerous studies have employed ML algorithms to forecast AP mortality rates (5), severity (6–8), complications (9), recurrence rates (10), as well as surgical or intervention strategies (7), with ML exhibiting robust reliability in these domains.

In recent years, ML algorithms and the prediction models based on them have generated significant interest among researchers. A growing body of evidence indicates that ML plays a crucial role in predicting acute pancreatitis diagnosis and prognosis. This review aims to offer an overview of the specific applications of ML in AP, with the expectation that artificial intelligence can furnish more evidence-based support for clinical practice in the future.

## 2 The role of ML in predicting AP mortality

According to the 2012 revision of Atlanta classification (RAC) (11), SAP accompanied by persistent organ failure carries a high mortality rate, ranging from 20 to 40% (1). When complicated by late-stage infections, the mortality rate becomes exceedingly high. Traditional scoring systems for predicting mortality are complex and limited. A systematic review revealed that the positive predictive values of the APACHE II score (AcutePhysiology and Chronic Health Evaluation score, widely used in the classification of critically ill patients and prognosis prediction, which can make a quantitative evaluation of the patient’s condition, a higher score indicates a more serious condition, a poorer prognosis, and a higher rate of mortality), Ranson score (one of the earliest scoring systems for predicting the severity of AP and is primarily used to predict the severity of biliary pancreatitis), and Glasgow criteria (emphasis on objective laboratory indicators, including 8 indicators, assessed in 48h of admission to the hospital) were only 69, 63, and 66% respectively (12). Although APACHE II provides the best predictive value for mortality, there is currently no single scoring system that can reliably predict the mortality rate of acute pancreatitis. Therefore, in recent years, numerous early prediction models based on ML algorithms have been developed. These models offer valuable insights for early intervention and potentially reducing the mortality rate of SAP.

Ding et al. initially developed an artificial neural network (ANN) model using the MIMIC-III database, achieving an area under the receiver operating characteristic curve (AUC) of 0.769, which outperformed logistic regression with an AUC of 0.607, Ranson score with 0.652, and SOFA score with 0.401 in predicting in-hospital mortality rate for AP patients (13). The ANN model demonstrated superior overall performance and early-stage risk stratification capability for high-risk AP patients. Building on this, Ren et al. identified 856 AP admitted to the intensive care unit (ICU) from the MIMIC-IV database and developed 9 ML models. Among these, they selected the Gaussian naive Bayes (GNB) model, which demonstrated an AUC, accuracy, sensitivity, and specificity of 0.840, 0.787, 0.839, and 0.792 respectively—making it the most effective among all models tested (14). The GNB model’s ability to identify high mortality risk in AP patients admitted to the ICU was further validated using an external database. Similarly, ML models, especially support vector machine (SVM) models, play a crucial role in predicting 28-day all-cause mortality in patients with SAP and analyzing their risk factors (15). The superior attributes of these models compared to traditional scoring systems enhance their effectiveness in early identification of SAP patients and reducing their mortality risk.

However, when it comes to specific causal diagnosis of AP for predicting mortality rates, the predictive capability of gradient boosting machine (GBM) machine learning models appears to be insignificant. Luthra collected 97,027 patients with biliary pancreatitis from the Nationwide Readmission Database over a 4-year period and compared the differences in predicting AP patient mortality between the GBM machine learning model and multivariate logistic regression analysis, finding that the GBM machine learning model had a higher positive predictive value (47.3% vs 35.9%) and lower sensitivity (40.1% vs 46.7%) (16). Therefore, he believes that in a large national database, traditional analysis and GBM machine learning model are comparable and not inferior, and the application of machine learning in managing database-based models for predicting hospital mortality due to common disease states is limited. It is worth noting that after statistical analysis, he found that the inpatient mortality rate of biliary pancreatitis was 0.97%, and hospital stay, age, SAP, patient income quartile, and sepsis were determined as the main predictors of mortality in biliary pancreatitis after it was determined.

## 3 The role of ML in predicting AP severity

A recent study in Japan showed that the mortality rate of SAP is about 16.7% (17), and early identification and personalized precision treatment can reduce the mortality rate of SAP. Previous studies have shown that precision treatment within 48 h of admission can significantly reduce mortality from SAP (18).

Due to the severity of SAP, high mortality rate, and association with organ failure, early identification and intervention of SAP patients are crucial. However, traditional scoring systems often require more than 24 h to perform and have limited accuracy. To address this, Luo et al. constructed and compared the predictive performance of five different ML models in training and validation cohorts, concluding that the random forest (RF) model performed the best and could be used to guide treatment and improve clinical outcomes (19). The AUC, accuracy, sensitivity, specificity, positive predictive value (PPV), and negative predictive value (NPV) of the RF model were 0.961, 86.0, 90.0, 81.5, 84.4, and 88.0% in the training cohort, and 0.969, 90.1, 88.6, 91.5, 91.2, and 89.0% in the validation cohort, which were significantly higher than those of other scoring systems (20, 21), the RF model has a higher accuracy in predicting SAP in the early stages of AP (22). Similarly, after developing and comparing different ML prediction models in terms of their effectiveness in predicting the severity of AP, Rahul et al. concluded that the extreme gradient boosting (XGBoost) model showed the best performance in predicting SAP, which can accurately predict SAP at an early stage and provide assistance to clinicians in identifying and intervening in SAP earlier (8, 23, 24). XGBoost is a machine learning technique that integrates regression tree gradient lifting methods and has gained widespread recognition in the machine learning literature (25–27), data mining challenges, and disease outcome prediction. Given its ability to predict SAP by combining imaging findings and clinical indicators, as well as its capacity to effectively handle missing values commonly encountered in clinical settings (8), early classification and identification of AP can provide valuable guidance for improved integration of medical resources.

## 4 The role of ML in predicting AP complications

### 4.1 Organ failure (OF)

Approximately 20% of patients develop organ failure in AP (28), and the presence of persistent organ dysfunction is a key factor in distinguishing between MAP, MSAP and SAP. Once OF occurs, the mortality rate can be as high as 30% (29), while also increasing the risk of infected pancreatic necrosis. Therefore, early identification of AP complicated by OF has a crucial impact on the emergency management of AP patients and plays a vital role in improving survival rates.

Four studies designed ML models to predict OF (9, 30–32). Qiu established models based on SVM, logistic regression (LR), and ANN to predict multiple organ failure (MOF) (30). The area under receiver operating characteristic curve (AUROC) values of these three models were not significantly different at 0.840, 0.832, and 0.834 respectively. Additionally, there was no significant difference in the AUROC compared to the traditional APACHE II score with an AUC value of 0.814 where *P* > 0.05. He believes that the three ML models can all be effective prognostic tools for predicting MOF in MSAP and SAP, and recommends using ANN, which only requires hematocrit, kinetic-time, IL-6, and creatinine as four common parameters. A multicenter cohort study employed complete blood count, serum biochemical markers, and coagulation indicators to develop 6 ML-based algorithm models for predicting MOF (9). Among these, the Adaptive Boosting algorithm (AdaBoost) exhibited superior predictive performance with an AUC of 0.826, sensitivity of 0.805, and specificity of 0.733. IL-6, creatinine, and kinetic time in coagulation indicators were identified as the three most significant independent variables, and monitoring these features can aid in preventing AP-related MOF. Numerous studies have indicated that the conventional use of ANN models is superior to APACHE II scores and LR models in predicting disease severity, MOF, and mortality, and the ANN models can accurately classify 96.2% of patients (31, 33). Lin et al. collected data from 314 Hyperlipidemic acute pancreatitis (HLAP) patients and established LR, NB (Naive Bayes), KNN (K-Nearest Neighbors), DT (Decision Tree) and RF models (32). The AUC values were 0.838, 0.824, 0.853, 0.897, and 0.915 respectively, all significantly higher than those of traditional prediction scoring systems. Among them, the RF model exhibited the highest predictive AUC for OF in HLAP patients with a sensitivity of 0.828 and accuracy of 0.814 among the 5 models tested. They concluded that the RF model outperforms other models as well as clinical scoring systems in predicting the occurrence of OF in HLAP patients and is beneficial for early intervention in high-risk HLAP patients for OF prevention.

AP-related OF mainly involves respiratory, circulatory, and renal failure. Some retrospective clinical analyses have confirmed the role of ML in acute kidney injury (AKI) associated with AP (34–36). Zhang et al. developed an automated machine learning (AutoML) algorithm prediction model that intelligently selects from a range of algorithms and hyperparameters to tailor models for specific datasets (34), enabling early prediction of AKI in AP patients. It demonstrates superior performance compared to traditional LR, requiring less time and achieving higher accuracy, thus significantly improving work efficiency. This warrants its clinical application and promotion. Lin et al. extracted data from the MIMIC-IV database to build a predictive ML model for SAP-AKI using 1,235 cases of SAP patients (35). The models included GBM, GLM, KNN, NB, ANN, RF, and SVM with AUC values of 0.814, 0.812, 0.671, 0.812, 0.688, 0.809 and 0.810 respectively. This highlights the significant role of GBM in predicting SAP-AKI and can assist clinical practitioners in identifying high-risk patients and intervening promptly to reduce mortality rates in intensive care units. It is also worth noting that systemic inflammatory response is inherently associated with the process of AKI and may be caused by local inflammation within renal tissues (37).

Acute respiratory distress syndrome (ARDS) is a common complication of AP, with approximately 30% of SAP patients developing ARDS (38), resulting in a mortality rate of up to 37% (28). Two retrospective analysis studies exploring ML models for AP-associated ARDS have yielded positive results (39, 40), successfully establishing predictive models based on ML. Compared with other models, the Bayesian Classifier (BC) model achieving the highest AUC at 0.891 and demonstrating the best predictive performance (39). The Ensemble Decision Trees (EDT) showed good predictive capabilities, with the highest accuracy (0.891) and precision (0.800). It is noteworthy that lower PaO_2_ and Ca^2+^ levels upon admission, as well as elevated CRP, Procalcitonin, Lactic Acid, Neutrophil-Lymphocyte Ratio, White Blood Cell Count, and Amylase levels are significantly associated with an increased risk of developing ARDS in AP patients; among these features, PaO_2_ is identified as the most important predictor.

### 4.2 Sepsis

ML techniques also demonstrate significant advantages in predicting and evaluating septic shock. In a large retrospective cohort study (41), 1,672 AP from the MIMIC III and MIMIC IV databases were selected to construct six ML models, including SVM, KNN, Multilayer Perceptron (MLP), LR, Gradient Boosting Decision Tree (GBDT), and AdaBoost. The GBDT model demonstrated superior performance in predicting sepsis among AP patients with an AUC of 0.985 on the test set, outperforming LR, Systemic Inflammatory Response Syndrome (SIRS) score, Bedside Index for Severity in Acute Pancreatitis (BISAP) score, Sequential Organ Failure Assessment (SOFA) score, quick SOFA (qSOFA), and APACHE II scores in sepsis prediction. Similarly, another retrospective study data established multiple ML models for early prediction of septic shock in AP with sepsis (42), with the final auto-encoder (AE) model achieving the highest AUC on the validation set (AUC 0.900, accuracy 0.868), while the AUC on the test set was 0.879 and the accuracy was 0.790. The AE model performed better than traditional scoring systems in predicting septic shock in AP with sepsis within 28 days after admission.

## 5 The role of ML in predicting AP recurrence rate

Recurrent acute pancreatitis (RAP) is defined as a history of at least two episodes of AP with no evidence of pancreatic tissue or functional abnormalities during the remission period. It represents a distinct subtype of pancreatitis, and statistics indicate that 17–22% of diagnosed AP patients will experience recurrence (43). RAP serves as a significant risk factor for the development of chronic pancreatitis (CP), with up to 36% of RAP patients ultimately progressing to CP (44). CP is often accompanied by comorbidities such as diabetes, malnutrition, steatorrhea, and weight loss. Long-term follow-up studies have revealed that 1.3% of CP patients may progress to pancreatic cancer over an 8-year period (45), significantly impacting their quality of life and prognosis. Therefore, early identification and timely intervention for individuals at risk for developing RAP following an episode of acute pancreatitis may mitigate the incidence rates of both RAP and pancreatic cancer while enhancing long-term quality of life.

Radiomics is an emerging field that optimizes existing imaging resources to extract high-throughput quantitative features from medical images (46, 47). These features are further analyzed using predefined algorithms to develop models for clinical decision-making. Currently, radiomics has been widely applied in the precise analysis of tumors and their metastases (46).

ML models based on radiomics research for predicting RAP are currently underutilized in clinical practice. Two retrospective analysis studies have confirmed the role of SVM models in predicting and distinguishing RAP (10, 48). The SVM model demonstrates a significantly higher AUC than traditional clinical models (0.941 vs. 0.712, *p* = 0.000), with similar conclusions observed in the validation dataset (0.929 vs. 0.671, *p* = 0.000) (48). The SVM model constructed using radiomic features can effectively differentiate between patients with functional abdominal pain, RAP, and CP, achieving an overall average accuracy of 82.1%. For patients diagnosed with RAP solely based on symptoms of abdominal pain and laboratory values or those for whom imaging studies during AP episodes are unavailable, radiomics may serve as a valuable diagnostic adjunct (10).

## 6 The role of ML in predicting AP surgical intervention strategy

Infected pancreatic necrosis (IPN) is the most severe local complication in the late stage of AP. Once IPN occurs, it indicates SAP, with a mortality rate as high as 30% (49). Treatment often involves a series of surgical debridement procedures known as “Step-up” strategies, including percutaneous catheter drainage (PCD), endoscopic transgastric necrosectomy, video-assisted minimally invasive surgery, and open surgery (50). There has been significant debate regarding the timing of surgical intervention for IPN. Research suggests that early surgery results in a mortality rate exceeding 50% (51), while delaying surgery until 4 weeks after the onset of IPN can reduce both complications and mortality rates (52). With the advancement of modern minimally invasive techniques, early endoscopic drainage during the course of AP has also proven to be safe and effective (53, 54). Early, timely, and accurate prediction of IPN occurrence and determination of the optimal timing for surgical intervention are crucial factors guiding subsequent treatment decisions.

Lan et al. included 223 patients with IPN who underwent surgical treatment for AP (5). They classified IPN patients based on whether the surgery was performed within 4 weeks using LR, SVM, and RF models. The RF model demonstrated a higher classification accuracy (0.80) compared to SVM (0.78) and LR (0.71). Additionally, they identified IL-6, infectious necrosis, fever, and CRP as key factors in determining the timing of surgical intervention for IPN patients. The ML model can effectively predict the optimal timing for surgical intervention in IPN, providing valuable guidance for clinicians in developing personalized surgical strategies for IPN patients.

Another large-scale retrospective clinical study involving 15,813 patients with AP has developed a novel ML model based on recurrent neural network (RNN) to predict the timing of surgical intervention for IPN (55). This model, known as Phased Long Short-Term Memory (Phased-LSTM), achieved an AUC greater than 0.70 and demonstrated stronger interpretability, making it suitable for predicting the optimal timing for surgery. The developed model visualizes specific surgical timings and changes in laboratory indicators from onset to discharge for AP patients, enabling comprehensive monitoring of patients with necrotizing pancreatitis throughout their hospitalization. Due to the ability of LSTM to forget and update long-term states, its performance surpassed that of SVM and RF, highlighting the advantages of time series models in handling temporal data.

## 7 Discussion

With the improvement in living standards, the incidence of AP has been increasing annually in recent years, with a rise of approximately 2–5% per year (56). Concurrently, the proportion of SAP is also rising. SAP is closely associated with multiple organ failure and has a high mortality and recurrence rate. Once the condition progresses to IPN and OF, the mortality rate can reach up to 30% (1). Therefore, early prediction of the severity of AP, the occurrence of complications, and the timing of intervention is crucial for clinical decision-making and timely intervention. However, traditional clinical prediction models, which are often based on multivariable analysis, are challenging to construct within 24h widespread clinical application. Consequently, it is imperative to develop a simple, effective, and clinically implementable model for early prediction of AP progression.

Artificial Intelligence (AI) encompasses a range of subfields within computer science. In recent years, advancements in algorithms such as ML, statistical learning, deep learning, and cognitive computing have played a pivotal role in the diagnosis and treatment of diseases such as sepsis and cancer (4, 57). ML, a subset of AI, is a burgeoning interdisciplinary field that integrates statistics, computer science, and other areas. It is not only used for text mining and classification in computer science, but is also increasingly applied in clinical practice. Various ML algorithm models for disease prediction and diagnosis have been developed based on AI technologies and are now widely accepted in the medical field. Recently, ML has begun to be applied to areas such as the severity of AP, complications, recurrence rates, organ dysfunction, and the timing of surgical intervention. This review focuses on recent advancements in the application of ML models in the context of AP (refer to Table 1).

**TABLE 1:** The clinical application of machine learning in acute pancreatitis.

References	Disease	Sample size	ML-based model	Contrast model	AUC (95%CI)
Ding et al. (13)	Mortality	337	ANN	LR	0.769
Ren et al. (14)	Mortality	856	GNB	XGBoost, RF, SVM, et al.	0.840
Cai et al. (15)	Mortality	534	SVM	LR, XGBoost, RF, et al.	0.877
Anjuli et al. (16)	Mortality	97,027	GBM	LR	0.96
Qiu et al. (30)	Multiple Organ Failure	263	SVM	LR, ANN	0.840
Zhang et al. (34)	Acute kidney Injury	437	AutoML	LR, DL	0.963
Liu et al. (41)	Sepsis	1,672	GBDT	LR, SVM	0.985
Zhang et al. (39)	Acute Respiratory Distress Syndrome	460	BC	SVM, EDTs	0.891
Xia et al. (42)	Septic Shock	604	AE	SVM, RF, AdaBoost, et al.	0.900
Xu et al. (9)	Multiple Organ Failure	455	AdaBoost	LR, et al.	0.826
Lin et al. (32)	Organ Failure	314	RF	LR, RF, et al.	0.915
Lin et al. (32)	Acute kidney Injury	667	GBM	NB, KNN, et al.	0.814
Chen et al. (48)	Recurrence	389	LR	SVM	0.941
Rahul et al. (8)	SAP	61,894	XGBoost	LR, ANN	0.921
Zhou et al. (23)	Severity of AP	441	XGBoost	LR, SVM, DT, RF	0.906
Lan et al. (5)	Surgical Intervention Strategy	223	RF	LR, SVM	0.78
Luo et al. (55)	Surgical Intervention Strategy	15,813	RNN	NA	0.70

ANN, artificial neural networks; LR, logistic regression; DL, deep learning; BC, Bayesian Classifier; EDTs, Ensembles of Decision Trees; AE, auto-encoder; AB, AdaBoost; RF, random forest; NB, naive Bayes; KNN, k-nearest neighbors; XGBoost, extreme gradient boosting model; RF, random forest; RNN, recurrent neural network.

We have observed that the majority of current ML models do not account for several important factors, including the etiology of AP and the stratification of severity. Additionally, most of the data utilized are retrospective, although these models have been validated on test and validation sets, their reliability still requires confirmation through clinical practice. Many studies are single-center with small sample sizes and lack external validation. Most research focuses on binary classification of AP into SAP and non-SAP. To date, there have been no ML models that provide accurate prognostication based on the 2012 Atlanta classification, which includes SAP, MSAP, and mild acute pancreatitis (MAP). Notably, most ML models remain limited to predicting traditional severity and complications, with a significant gap in predictive models for recurrence rates, optimal timing for surgery, pancreatic necrosis accumulation, and local complications such as infectious pancreatic necrosis. Future research should address these areas.

## 8 Conclusion

In conclusion, ML has proven to be an excellent predictor of mortality, severity, complications, recurrence, organ dysfunction, and timing of surgical intervention in acute pancreatitis, and is superior to traditional scoring systems such as the APACHE II score, the BISAP score, the SOFA score, and other traditional systems. However, much more prospective clinical studies are needed to validate this idea.
